# A minority of proliferating human CD4^+^ T cells in antigen-driven proliferation assays are antigen specific

**DOI:** 10.3389/fimmu.2024.1491616

**Published:** 2024-10-28

**Authors:** Pushpak Bhattacharjee, Miha Pakusch, Matthew Lacorcia, Chris Y. Chiu, Xin Liu, Eleonora Tresoldi, Abby Foster, Laura King, Fergus J. Cameron, Stuart I. Mannering

**Affiliations:** ^1^ Immunology and Diabetes Unit, St. Vincent’s Institute of Medical Research, Melbourne, VIC, Australia; ^2^ Victorian Centre for Functional Genomics, Melbourne, VIC, Australia; ^3^ Department of Endocrinology and Diabetes, Royal Children’s Hospital, Melbourne, VIC, Australia; ^4^ Diabetes Research Group, Murdoch Children’s Research Institute, Melbourne, VIC, Australia; ^5^ Department of Paediatrics, University of Melbourne, Melbourne, VIC, Australia; ^6^ Department of Medicine, University of Melbourne, St. Vincent’s Hospital, Melbourne, VIC, Australia

**Keywords:** clonal expansion, proliferation, CD4^+^ T cell, autoimmunity, antigen specific T cell, C-peptide, tetanus toxoid, CFSE assay

## Abstract

Antigen-driven T-cell proliferation is often measured using fluorescent dye dilution assays, such as the CFSE-based proliferation assay. Dye dilution assays have been powerful tools to detect human CD4^+^ T-cell responses, particularly against autoantigens. However, it is not known how many cells within the proliferating population are specific for the stimulating antigen. Here we determined the frequency of CD4^+^ T cells specific for the stimulating antigen within the antigen-responsive population of CFSE-based proliferation assays. We compared CD4^+^ T-cell responses to a type 1 diabetes autoantigen (proinsulin C-peptide) and to a vaccine antigen (tetanus toxoid). The TCRs expressed by antigen-responsive CD4^+^ T cells were sequenced, and their antigen specificity was tested functionally by expressing them in a reporter T-cell line. Responses to C-peptide were weak, but detectable, in PBMC from individuals with T1D, whereas responses to tetanus toxoid were much stronger. The frequency of antigen-specific CD4^+^ T cells correlated with the strength of the response to antigen in the proliferation assay. However, antigen-specific CD4^+^ T cells were rare among antigen-responsive CD4^+^ T cells. For C-peptide, an average frequency of 7.5% (1%–11%, *n* = 4) of antigen-responsive CD4^+^ T cells were confirmed to be antigen specific. In the tetanus-toxoid-stimulated cultures, on average, 45% (16%–78%, *n* = 5) of the antigen-responsive CD4^+^ T cells were tetanus toxoid specific. These data show that antigen-specific CD4^+^ T cells are a minority of the cells that proliferate in response to antigen and have important implications for *in vitro* CD4^+^ T-cell proliferation assays.

## 1 Introduction

The capacity to proliferate in response to antigen is a hallmark of adaptive immunity (1). CD4^+^ T cells “recognize” antigen in the form of peptides presented in association with HLA, or MHC in the mouse, class II on the surface of professional antigen-presenting cells (2). Upon antigen recognition, in the context of the appropriate costimulatory and cytokine signals, many CD4^+^T cells proliferate. For this reason, measuring T-cell proliferation has long been used to analyze human-antigen-specific T-cell responses *in vitro* (3). Initially, ^3^H-thymidine incorporation was used to quantify antigen-stimulated proliferation (4). More recently, dye dilution assays, which use stable fluorescent dyes, such as CFSE (5), distinguish cells that have proliferated from resting cells. This principle has been used to develop the CFSE-based proliferation assay (6, 7). This assay works by using flow cytometry to count the number of cells that have proliferated in response to an antigen compared to background proliferation in the absence of antigen. There are now many available dyes that work similarly to CFSE that have been used in dye dilution assay (8). Here we focus on CFSE which is a representative of other dye dilution assays. The great advantage of the CFSE-based proliferation assay and dye dilution assays in general is that they are very sensitive. They have been particularly useful to detect CD4^+^ T-cell responses to antigens that are weakly immunogenic and/or when the frequency of antigen-responsive cells is low—for example, in allergy (9) or autoimmunity (10, 11). In addition, since the cells remain viable, dye dilution assays allow the responding cells to be cloned (12) or phenotypically evaluated (13, 14).

The strength of a response to an antigen in a human dye dilution assay can be quantified in two ways: either as a percentage of total cells (15) or, as we prefer, as a ratio, which we call the cell division index or CDI (6). Unlike transgenic murine T cells (16), polyclonal human cells do not give discrete peaks, which makes counting divisions more difficult in assays using human cells. The CDI is the ratio of the number of cells that have proliferated with an antigen against the number of cells that proliferated in the absence of an antigen (6). We have assumed that if there are threefold more proliferating cells with an antigen than without, that is CDI = 3.0, then the majority (~66%) of the antigen-responsive cells are specific for the antigen added to the assay. However, cloning CFSE^dim^, antigen-responsive CD4^+^ T cells (12) showed that CD4^+^ T-cell clones specific for the stimulating antigen could be isolated, but these clones were not as abundant as expected within the CFSE^dim^ population. However, from these cloning experiments, we could not infer the frequency of antigen-specific CD4^+^ T cells within the CFSE^dim^ population because antigen-specific CD4^+^ T cells could have been lost during the cloning, expansion, and screening process. Hence, the frequency of antigen-specific T cells within the CFSE^dim^ population remained unclear.

Since the CFSE-based proliferation assay and other dye dilution assays are commonly used to detect responses to weak antigens, including autoantigens, we wanted to determine the antigen specificity of CD4^+^ T cells that respond to these antigens. We have previously shown that C-peptide, which is produced when proinsulin is converted to insulin, is an important antigen in human type 1 diabetes (T1D) (10, 11). Hence, we set out to determine how faithfully the CFSE-based proliferation assay reported the proliferation of C-peptide-specific CD4^+^T cells. For comparison, we also investigated the responses to a vaccine antigen—tetanus toxoid. The antigen specificity of the responding cells was determined by sequencing the TCR genes expressed by antigen-responsive (CD4^+^ and CFSE^dim^) cells and expressing these TCRs in a reporter Jurkat T-cell line, allowing us to functionally test the antigen specificity of the population of CD4^+^ T cells that proliferated in response to each antigen. Surprisingly, we found that a minority of the antigen-responsive (CFSE^dim^) CD4^+^ T cells were antigen specific.

## 2 Methods

### 2.1 Subjects and ethical approval

Ethical approval was given by St. Vincent’s Hospital HREC (2023/PID00247, HREC-A 161.15) and Southern Health/Royal Children’s Hospital (12185B). All participants provided written informed consent.

### 2.2 Antigen-responsive CD4^+^ T-cell isolation and analysis

The CFSE (5,6-carboxylfluorescein diacetate succinimidyl ester) proliferation assays were performed with PBMC from individuals with T1D in the presence of full-length C-peptide (PI_33–63_ at 10 μM), tetanus toxoid (0.33 LfU/mL) or no antigen, as described previously (6). Briefly, the CFSE-assay-responding CD4^+^ T cells were barcoded to distinguish cells responding to the different antigens. Responding CD4^+^ and CFSE^dim^ cells were FACS-sorted, and cells from the same donors were pooled. The TRA and TRB sequences were determined by 10X single-cell sequencing. Cells expressing one TRBV beta and up to two TRAV genes were considered to be a paired TCR clonotype. Up to 30 most abundant clonotypes for each antigen were assembled, using custom R script, for DNA synthesis and cloning (see **Supplementary Methods** for a detailed method description).

### 2.3 Generation of parental JNL

Starting with Jurkat E6.1, we generated a subline that was TCR-deficient CD4^+^ and had a luciferase reporter gene knocked immediately following the IL-2 promoter. CRISPR/Cas9 was used to generate a subline which did not express TRA, TRB and CD4. To facilitate the analysis of TCRs from CD4^+^ T cells, CD4 was re-introduced into the Jurkat cells by lentiviral transduction. A NanoLuciferase reporter was then knocked into the IL-2 locus. This parental luciferase reporter Jurkat cell line (Jurkat NanoLuciferase- referred to a JNL) was then transduced with the lentiviral TRAV and TRBV constructs as described below.

### 2.4 Generation of T-cell avatars

TCR variable alpha and TCR beta genes were synthesized (IDT, Gene Universal) and cloned into a derivative of pRRLSIN (**Supplementary Table S4**) containing the TCR constant regions. Plasmids were extracted from growing *E. coli*, and the inserts were verified by sequencing. The JNL cells were transduced with lentivirus encoding TRAV and TRBV constructs for each TCR. TCR-expressing lentiviruses were generated by transfecting HEK293T cells with the appropriate paired TCR-expressing pRRLSIN plasmids and the packaging plasmids (pMDLg/pRRE, pRSV-Rev, and pMD2.g) using Lipofectamine 2000 (Invitrogen). After 24 h of transfection, a lentivirus-containing supernatant was used to transduce the JNL cells. The JNL cells were resuspended in filtered viral supernatant at 1 to 2 × 10^6^/mL with 5 μg/mL polybrene. Then, the JNL cells were centrifuged with the viral supernatant at 1,200 rpm (300 × *g*) for 60 min, then diluted 1:1 with fresh medium, and incubated overnight at 37°C, 5% CO_2_. The following anti-human mAbs were used for FACS staining: human anti-CD3-PE (UCHT1, BD Biosciences) and human anti-TCRαβ-AF647 (IP26, BioLegend). The cells were stained with the appropriate mAb in phosphate-buffered saline and 0.1% fetal bovine serum for 20 min on ice and then washed twice. Dead cells were excluded by using propidium iodide. TCR^+^ and CD3^+^ cells were purified by FACS or using REALease CD3 Microbead Kit, human (Miltenyi). All purified avatars were confirmed by using flow cytometry and were above 90% TCR^+^ and CD3^+^.

### 2.5 Screening TCR for antigen specificity

For TCR response assays, 1 × 10^4^ TCR expressing JNL cells were co-cultured with 1 × 10^4^ autologous Epstein–Barr virus (EBV)-transformed B cells and no antigen, anti-CD3 mAb (OKT3, WEHI), or antigen (C-peptide 10 μM or 0.33 LfU/mL tetanus toxoid) in triplicate in a 96-well plate (Nunc). After 24 h, NanoLuciferase was measured using the Nano-Glo Luciferase assay (Promega) according to the manufacturer’s instructions. Luminescence was measured on a Perkin Elmer Enspire plate reader. Responses are reported as the Δluciferase, which is calculated by subtracting the average background luciferase reading measured in the no-antigen control cultures from the responses measured in the other treatments.

### 2.6 Validation of T-cell responses with synthetic peptide

For the JNL assay for tetanus toxoid responses, autologous EBV cells were cultured overnight with tetanus toxoid before adding the T-cell avatars. For full length, C-peptide (PI_33-63_) (Genscript, or Mimotopes) was added to the EBV cells at the same time as the JNL cells. A T-cell avatar’s response to an antigen was measured by luciferase activity using Nano-GloLuciferase (Promega). Responses are expressed as delta (Δ) luciferase, calculated by subtracting the average luciferase in the “no antigen” cultures from the luciferase readings of cultures with an antigen. The minimum stimulatory concentration (MSC) of C-peptide was determined from the concentration of C-peptide required to stimulate a response above the background, which was set at Δluciferase of 2 × 10^4^ units. We calculated the proportion of antigen-specific CD4^+^ T cells by determining the percentage of antigen-specific clonotypes within the population of clonotypes analyzed.

### 2.7 Statistics

Graphing and statistical analysis were done using Prism 10.0.3. Statistical significance was determined using one-way ANOVA and corrected for multiple comparisons using Dunnett statistical hypothesis testing. The patients’ responses were compared using Mann–Whitney test, and statistical significance was defined as *p <*0.05 as shown in the figure legends.

## 3 Results

### 3.1 TCR sequencing of CD4^+^ T cells that proliferate in response to C-peptide or tetanus toxoid

**Figure 1:**
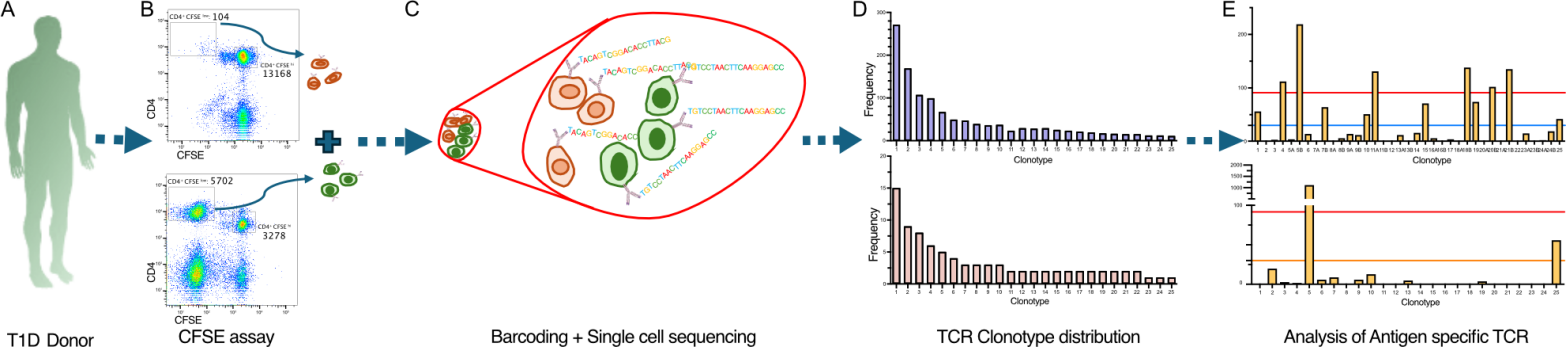
Workflow to identify antigen-specific CD4^+^ T cells in the CFSE-based proliferation assay. The workflow started from isolating PBMC from each donor, which were stained with CFSE and cultured with tetanus toxoid or C-peptide for 7 days **(A)**. Then, hash-tagging was used to mark cells of different antigen treatments. Using FACS, CFSE^dim^ CD4^+^ cells were sorted for different antigen treatments to isolate the antigen-responsive population **(B)**. These CFSE^dim^ CD4^+^ cells derived from different antigens were pooled and processed for single-cell TCR sequencing using 10X Genomics’ 5′ platform **(C)**. The first output from the TCR sequencing is represented by the TCR clonotype abundancy distribution plot **(D)**. Then, the most abundant paired TCR clonotypes were screened to identify antigen-specific TCRs among the antigen-responding population **(E)**.

**Figure 2:**
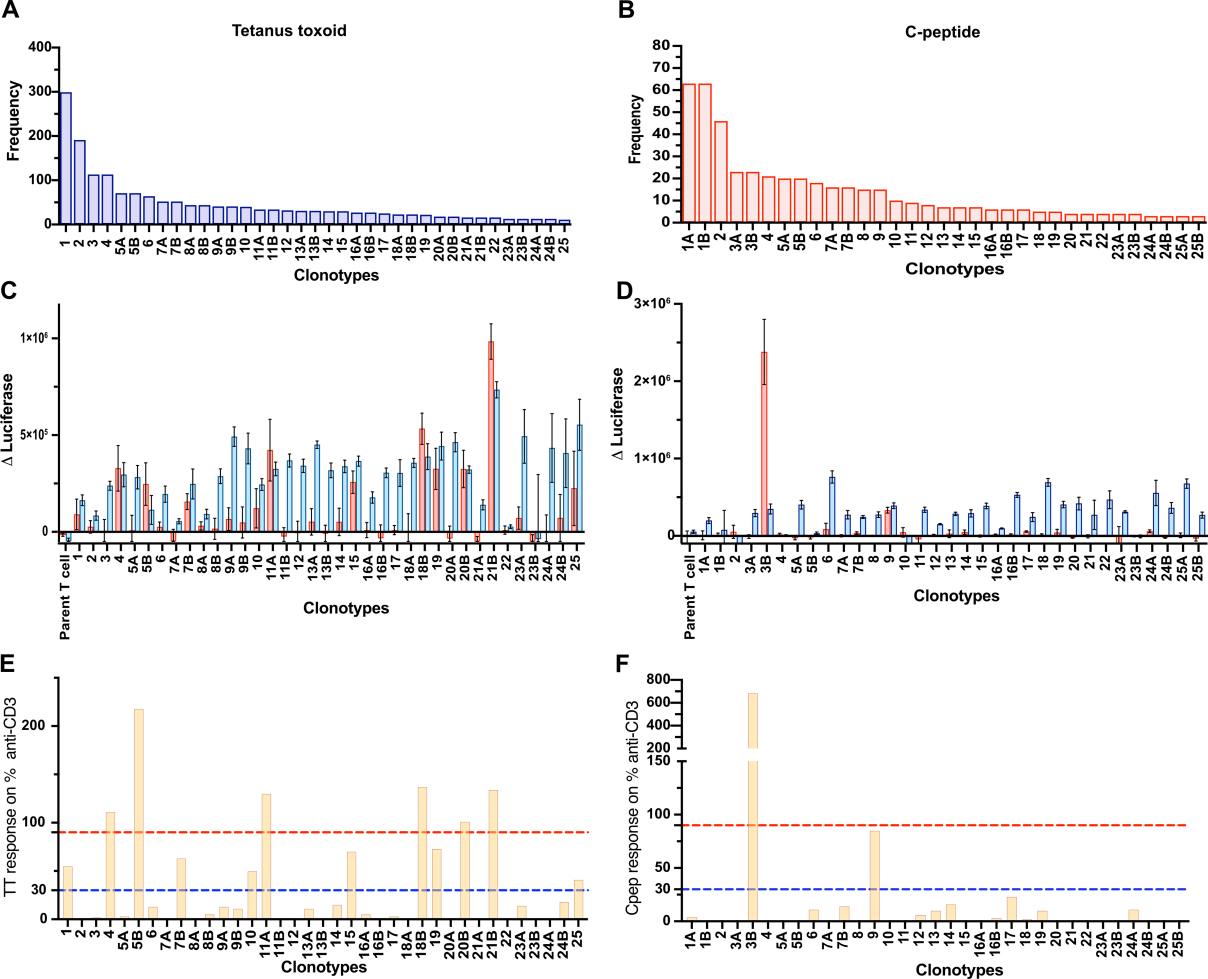
Screening TCRs for response to tetanus toxoid and C-peptide. Frequency distribution of the 25 most abundant TCR clonotypes derived from Donor 282T2’s antigen-responsive CD4^+^ T cells for tetanus toxoid **(A)** and C-peptide **(B)**. The TCR clonotypes are numbered according to their abundance in the antigen-responsive population (1–25, most to least abundant). TCR clonotypes that comprised two TRAVs are indicated as “A” and “B” after the clone number. JNL avatars expressing the most abundant ~25 TCR clonotypes were screened for antigen specificity. Responses to antigen tetanus toxoid **(C)** and C-peptide **(D)** are expressed as Δluciferase. The antigen-specific responses plotted for individual JNL avatars are shown as a percentage of their response to anti-CD3 for tetanus toxoid **(E)** and C-peptide **(F)**. Anti-CD3 responses to the TCR negative, parental JNL cells plus 3× standard deviation of the “nil antigen” were used as a threshold for a positive response. Responses >30% of the anti-CD3 response, represented by a blue dotted line, are weak responding clones, and those >90% of the response to anti-CD3, represented by a red dotted line, were considered to be strongly antigen-specific TCRs.

We set out to determine the number of functionally antigen-specific CD4^+^ T cells among the population of cells that proliferate *in vitro* in response to an antigen. An outline of the workflow is shown in **Figure 1**. The CFSE-based proliferation assay was performed using the T1D autoantigen, proinsulin C-peptide (10, 11), or the vaccine antigen tetanus toxoid. All assays, except 1, used fresh PBMC (**Supplementary Table S2**). The CD4^+^ T cells that had proliferated in response to an antigen were identified by their CFSE dilution. This CFSE^dim^ population was sorted by using flow cytometry (**Supplementary Figure S1**). The TCR genes expressed by C-peptide- and tetanus-toxoid-responsive CD4^+^ T cells were determined by single-cell sequencing. The paired TCR clonotype distribution patterns for each antigen were obtained from five subjects with T1D. As expected, the TCR clonotypes varied in abundance (**Supplementary Figure S2**). For C-peptide-responsive CD4^+^ T cells, the most abundant TCR clonotypes were expressed by 15 to 126 CD4^+^ T cells, whereas for responses to tetanus toxoid the most abundant TCR clonotypes were expressed by 13 to 329 of the CD4^+^ T cells sequenced from the five subjects. **Figure 2** shows the results from one donor, and the results for all donors are summarized in **Supplementary Figure S2** and **Supplementary Table S2**. However, the clonotype frequency fell quickly, such that many clonotypes were only expressed by a few cells for each antigen (**Supplementary Figure S2**). For C-peptide-responsive TCRs, we screened the top 25–30 most abundant clonotypes for their antigen specificity; however, for tetanus-toxoid-responsive TCR, we screened the top 11–25 most abundant clonotypes for their antigen specificity from each subject (**Figures 2A, B**).

### 3.2 Developing a screening method to analyze the antigen specificity of antigen-responsive human CD4^+^ T cells

We established a functional TCR screening assay to identify the antigen-specific TCRs derived from antigen-responsive CD4^+^ T cells isolated using the CFSE-based proliferation assay. Each TCR clonotype was expressed in Jurkat line which is CD4^+^ TCR deficient and has a nanoluciferase reporter driven by the IL-2 promoter, referred to as Jurkat NanoLuciferase (JNL). JNL cells transduced with TRA and TRB genes are referred to as T-cell avatars. The antigen specificity of each paired TCR was determined by measuring antigen-stimulated luciferase expression (**Figures 2C, D**). There were two TRA chains in 16% (37 out of 231) of the clonotypes tested. In these cases, both TRA chains were tested with the corresponding TRB. Responses to the antigen are expressed relative to anti-CD3 stimulated responses (**Figures 2E, F**). Overall, more than 93% (215 of 231) of T-cell avatars responded to anti-CD3 mAb, indicating that they expressed sufficient TCR/CD3 on their surface to give a detectable response in the screening assay. The strength of response to the antigen, relative to the anti-CD3 mAb response, was divided into three categories: (i) negative, <30% of the anti-CD3 mAb response; (ii) weakly positive, 30%–90% anti-CD3 mAb response, and (iii) strong, >90% anti-CD3 mAb response (**Figures 2E, F**).

### 3.3 Validating the antigen-specific CD4^+^ T-cell response

**Figure 3:**
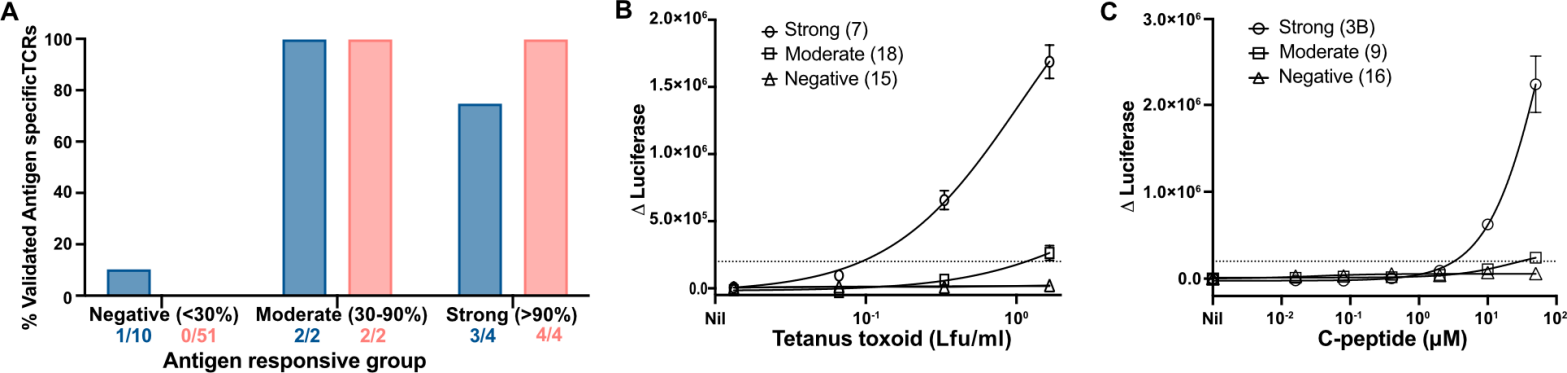
Validating the screening assays using T-cell avatar. **(A)** Antigen-responsive TCRs specific for tetanus toxoid or C-peptide derived from each of the three categories in the screening assay were validated using purified CD4^+^ T-cell avatar against their respective antigens **(A)**. The proportion of verified, antigen-specific TCRs is shown as the percentage of all TCRs tested for each category of response. Total TCRs tested for each category and for each antigen (C-peptide, shown as red bars, and tetanus toxoid, shown as a blue bar) were represented. Responses by JNL cells expressing tetanus-toxoid-specific TCRs **(B)** from Donor 312T4 and C-peptide **(C)**, from Donor 282T2, are shown for each category of response. A Δluciferase reading of 2 × 10^4^ RLU was determined as a threshold response and is shown here with a dotted line.

To validate the results of the TCR screening assay, we generated and purified T-cell avatars and analyzed their antigen specificity. A total of 57 avatars expressing TCRs from C-peptide stimulation experiments and 16 expressing TCRs from tetanus toxoid stimulation experiments were tested (**Figures 3A–C**). For C-peptide, there was 100% concordance between the screening assay and confirmation with purified T-cell avatars (**Figures 3A, C**). Of the 57 tested, 51 (89.4%) TCRs were confirmed to be unresponsive to up to 50 μM C-peptide. Two TCRs (3.5%) that were categorized as weakly responsive in the screening assay only responded weakly to the highest (50 μM) concentrations of the C-peptide tested (**Figures 3A, C**). In contrast, the four (7.0%) strongly positive TCRs were confirmed to respond vigorously at a minimum stimulatory concentration of C-peptide, which ranged between 0.4 and 10.0 μM (**Supplementary Table S3**). For tetanus-toxoid-responsive CD4^+^ T cells, 16 putative tetanus-toxoid-specific TCRs were analyzed in more detail (**Figure 3A**). The screening assay correctly identified nine of 10 TCR clonotypes as being unresponsive to tetanus toxoid at -90% accuracy (**Figures 3A, B**). One “negative” TCR responded very weakly to tetanus toxoid. Two weakly responsive TCRs identified in the screening assay were confirmed as being tetanus specific. The remaining four TCRs were identified in the screening assay as strongly responsive to tetanus toxoid. Three of these four were confirmed to be tetanus toxoid specific (75% accuracy), but one failed to respond (**Figure 3A**). Overall, the screening assay was 88% (14/16) accurate for tetanus toxoid and 100% accurate for C-peptide. The accuracy of the TCR screening assay is summarized in receiver–operator curves (ROC) (**Supplementary Figure S4**).

### 3.4 Analyzing the frequency of antigen-specific CD4^+^ T cells and their distribution

**Figure 4:**
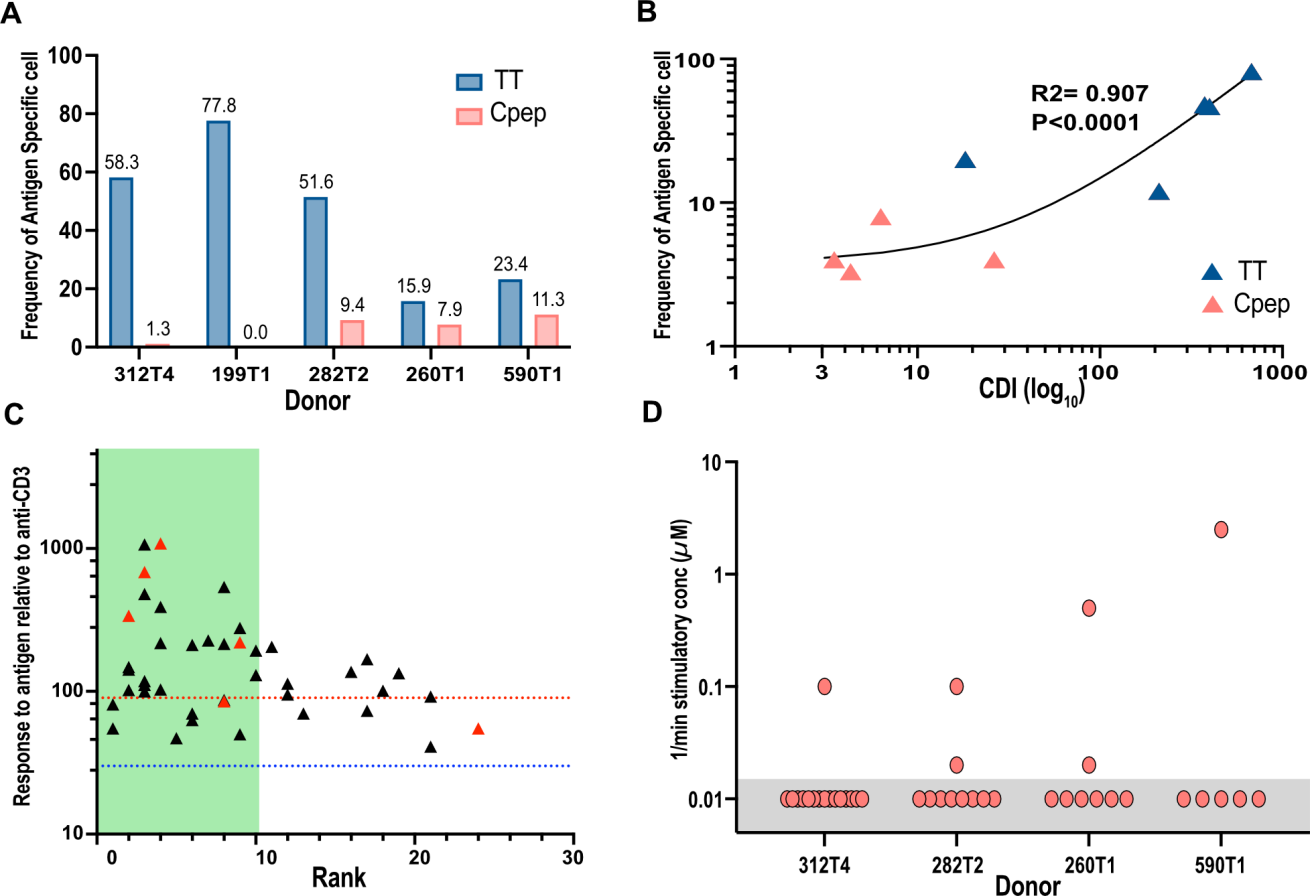
Analyzing the frequency of antigen-specific CD4^+^ T cells and their distribution. The proportion of tetanus-toxoid-specific (blue bars) or C-peptide-specific (red bars) T cells **(A)** from each donor is shown as a percentage of the TCRs tested. The numbers are the frequency for each bar. **(B)** The strength of the antigen-specific response in the original CFSE assay measured by the cell division index (CDI log_10_) for C-peptide (red) and TT (blue) was correlated with the proportion of antigen-specific TCR clone identified for each donor. *R* ^2^ is the correlation coefficient. **(C)** The magnitude of all antigen-specific CD4^+^ T-cell responses in the screening assays, relative to anti-CD3, is shown plotted against their individual clonotype rank. Responses to tetanus toxoid are represented by black triangles, and those of C-peptide are represented by red triangles. The blue dotted line indicates the threshold for a weak (30%) antigen-specific response, and the dotted red line represents the threshold (100%) for high-responding antigen-specific TCRs. The green-shaded area indicates the top-10-ranked clonotypes. **(D)** The inverse of the minimum stimulatory concentration) for C-peptide was plotted for all avatars tested for each donor. The C-peptide concentration used was between 0.016 and 50.0 μM. Any JNL avatars that did not respond to the highest concentration (50 μM) of C-peptide were arbitrarily given a value of 50 μM or 0.02. These responses are highlighted with a gray box.

Within the C-peptide-responsive CD4^+^ T cell population, we found that an average of 7.5% of the CD4^+^ T cells were (1%–11%, *n* = 4 individuals) C-peptide specific (**Figure 4A**). In total, we screened 130 TCRs for specificity for C-peptide. The specificity of all these TCRs was confirmed in titration experiments (**Supplementary Figure S3**). No C-peptide-specific CD4^+^ T cells were identified from one donor, perhaps because cryopreserved PBMCs were used for the CFSE assay (**Supplementary Table S2**). In total, we screened 101 tetanus-toxoid-responding TCRs from five donors (**Supplementary Table S2**), including the 16 which were validated as described above. The frequency of tetanus-toxoid-specific CD4^+^ T cells within the tetanus-responsive population was higher, averaging 45% (16%–76%, *n* = 5). The proportion of antigen-specific CD4^+^ T cells was significantly different between C-peptide and tetanus toxoid (two-tailed *t*-test, *p* = 0.0095). There was a positive correlation (*R* ^2^ = 0.907 and *p* < 0.0001) between the strength of the response to an antigen, measured as the CDI in the CFSE assay, and the frequency of validated antigen-specific clones (**Figure 4B**).

Sequencing allowed us to identify the most abundant clonotypes and relate this clonotype rank to antigen specificity and sensitivity (**Figure 4C**). Most, 70% (30 of 43), of the C-peptide- or tetanus-toxoid-specific TCRs were found within the top 10 most abundant clonotypes, while the remaining antigen-specific TCRs were evenly distributed among the less (11–25) abundant clonotypes (**Figure 4C**). An analysis of the C-peptide sensitivity of each TCR revealed a single highly sensitive TCR for each donor and one to two other antigen-specific TCRs with dramatically reduced antigen sensitivity (**Figure 4D**).

Overall, our data show that the antigen-specific CD4^+^ T cells comprise a minority of the CD4^+^ T cells that proliferate in response to the antigen *in vitro*. This phenomenon is particularly marked for C-peptide which stimulates a weak response.

## 4 Discussion

This, to our knowledge, is the first attempt to functionally define the antigen specificity of antigen-responsive CD4^+^ T cells in a dye dilution assay. Surprisingly, we found that the frequency of antigen-specific CD4^+^ T cells, within the antigen-responsive population, was very low.

Our data provides very useful insights into dye dilution assays. We found a clear positive correlation between the strength of the response in a CFSE assay and the number of verified antigen-specific CD4^+^ T cells (**Figure 4B**). Nonetheless, even when very strong responses were detected to tetanus toxoid, with CDIs commonly >300, the tetanus-toxoid-specific CD4^+^ T cells remained, on average, to be 45% of the antigen-responsive cells (**Figure 4A**). While the proportion varied between subjects, the maximum proportion of antigen-specific CD4^+^ T cells was 70% of the TCRs analyzed. Even when we see a very vigorous response to a recall antigen, the proportion of antigen-specific CD4^+^ T cells does not reach 100%. Our data shows that when a positive response is seen in a CFSE-based proliferation assay or a similar dye dilution assay, the actual number of antigen-specific cells is dramatically lower than previously assumed based on the ratio of the number of responding cells with and without antigen (CDI) or the percentage of antigen-responsive cells. This effect is particularly pronounced for antigens, like C-peptide, which stimulates a relatively weak response. However, while the number of C-peptide-specific CD4^+^ T cells was low, they were clearly present in all cases, except for one where we used PBMC that had been cryopreserved.

Proliferation assays have traditionally used an arbitrary cutoff to distinguish between a positive and a negative response. In the CFSE-based proliferation assay, we have considered CDI >3.0 to represent a positive response to an antigen. This was based on our experience with the CFSE-based proliferation assay followed by single-cell sorting/cloning (11, 12, 17–19). Now we confirmed that even when responses are weak, with CDI ~3.0, there are still detectable, albeit rare, antigen-specific T cells. Although we have not attempted to analyze antigen-responsive cells from CFSE assays with CDI <3.0, we expect that antigen-specific CD4^+^ T cells would not be detected. The AIM assay (20), which identifies antigen specific CD4^+^ T cells based on their upregulation of activation markers, is being used to identify and isolate beta-cell antigen-specific CD4^+^ T cells (21). Interestingly, in this assay, the antigen specificity of approximately a third of TCRs could not be defined (21). This highlights the challenges in detecting and measuring autoantigen-specific CD4^+^ T-cell responses in general and specifically beta-cell antigen-specific CD4^+^ T-cell responses.

Our detailed analysis of C-peptide-specific TCRs revealed a range of antigen sensitivities. While the number of antigen-specific TCRs that we could isolate and analyze was low, we found one very C-peptide-sensitive clonotype in most experiments. In some cases, we also found other TCRs which were C-peptide specific, but they required high (50 μM) concentrations of peptide to stimulate a response. This suggests that one or a few very antigen-sensitive CD4^+^ T cells may drive the proliferation of bystander T cells. Some of these bystanders may be weakly antigen specific. We have deliberately set a broad definition of antigen-specific TCRs, including those that respond to even weak to high concentrations of antigen. If we set a more stringent definition of antigen specificity, that is, responses to a lower concentration of antigen, we would find that only a single TCR would be antigen specific in each donor tested. Thus, our analysis may have over-estimated the frequency of C-peptide-specific TCRs. In any case, our conclusion that verified that antigen-specific CD4^+^ T cells are rare within the antigen-responsive population holds true.

The antigen specificity of the CD4^+^ T cells that are not specific for the stimulating antigen is currently unknown. We suggest that these cells are specific for commonly encountered viral, bacterial, or fungal antigens. These putative microbe-specific memory CD4^+^ T cells are relatively common in PBMC (22) and may proliferate in response to the cytokines produced by the relatively few CD4^+^ T cells specific for the stimulating antigen (23). We used the TCR databases VDJdb and MacPAS to predict the potential epitopes/antigens recognized by the bystander TCRs based on their CDR3β amino acid sequences. From this analysis, we identified approximately 20 candidate epitopes. We synthesized and tested peptides with the sequence of these predicted epitopes, but we did not find any that stimulated our bystander TCRs (data not shown). We did not attempt a more intensive approach to identify the antigens recognized by the bystander T cells because the number of potential antigens makes this task unfeasible. However, our clonotype analysis shows that these proliferating bystander cells are capable of proliferating very rapidly. We assume that their cognate antigens are not present in the *in vitro* cultures, but we cannot rigorously exclude the possibility that there are trace quantities of microbial antigens present in the samples that do not stimulate appreciable proliferation in the absence of the addition of an antigenic stimulus.

The strength of our approach was that we detected TCRs that responded to any epitope derived from the stimulating antigen, C-peptide and tetanus toxoid, in these experiments. This allowed us to directly compare the responses to an autoantigen and a vaccine antigen in the same individuals at the same time. By using autologous EBV-transformed B cells, we eliminated the possibility that responses would be overlooked because the restricting HLA allomorph was not present. Our TCR screening assay incorporated anti-CD3 as a positive control to confirm that the TCR was expressed and functional in the cells being tested. This allowed us to categorize responses into negative, weak, or strong. The advantage of single-cell sequencing is that it gives a clonotype distribution for each TCR pair. This, in turn, gives insights into the size of the clonal burst and how this relates to the antigen-stimulating antigen.

Our study has some weaknesses. We have limited our analysis to the top 11–30 most abundant TCR clonotypes for each antigen. It remains possible that we have not identified antigen-specific CD4^+^ T cells that are present within the lower clonotype frequencies. However, this is very unlikely because we did not detect more antigen-specific T cells at the lower end of the clonotype distribution curve (**Figure 4C**). We did not observe a clear decline in the frequency of antigen-specific CD4^+^ T cells that are in the lower clonotype ranks either. Nonetheless, the more antigen-sensitive and antigen-specific CD4^+^ T cells were more frequent within the top 10 most abundant clonotypes (**Figure 4C**). In most of our screening assays, we used one or two antigen concentrations. We cannot exclude the possibility that we did not detect antigen-specific T cells that required a high concentration of antigen. We feel again that this is unlikely because we could detect C-peptide-specific CD4^+^ T cells with EC_50_ from 2.0 to >50.0 μM. Some TCRs respond very weakly to an antigen, suggesting that we have identified all cells with some degree of antigen specificity. Finally, our work has used PBMC from individuals with T1D. We chose these subjects because we were interested in analyzing CD4^+^ T-cell responses to C-peptide which consistently stimulates a weak response in the CFSE-based proliferation assay (11). While we believe that it is very unlikely, we cannot exclude the possibility that different results would be obtained if we used PBMCs from individuals without T1D.

In conclusion, our data shows that CD4^+^ T cells specific for the stimulating antigen are in a minority of the cells that proliferate *in vitro*. While our data confirms that antigen-specific CD4^+^ T cells are present, it indicates that they are present at dramatically lower frequencies than previously appreciated. These findings will guide the interpretation of the results of dye dilution assays.

## Data Availability

The TCR sequence used in this article will be made available by the authors, without undue reservation.
