# Supplementary material for: A minority of proliferating human CD4+ T cells in antigen-driven proliferation assays are antigen specific
Source: Front Immunol. 2024 Oct 28;15:1491616. doi: 10.3389/fimmu.2024.1491616 (PMC11550966; doi:10.3389/fimmu.2024.1491616)
Supplement: Supplementary file 1 [file DataSheet1.docx]

**Supplementary Materials**

**A minority of proliferating human CD4^+^ T cells in antigen-driven proliferation assays are antigen specific**

Pushpak Bhattacharjee, Miha Pakusch, Matthew Lacorcia, Chris Y. Chiu, Xin Liu, Eleonora Tresoldi, Abby Foster, Laura King, Fergus J. Cameron, Stuart I. Mannering

**Supplementary Methods**

**Supplementary Methods 1:** The CFSE-based proliferation assay and 10X analysis

**Supplementary Methods 2:** TCR analysis

**Supplementary Methods 3:** Lentiviral constructs for expressing TCR genes

**Supplementary Tables**

**Supplementary Table 1:** Demographics and HLA of T1D blood donors.

**Supplementary Table 2:** TCR sequence of C-peptide specific CD4^+^T cell clones.

**Supplementary Table 3:** Summary of CFSE assay responses and TCR antigen specificity screening.

**Supplementary Table 4.** Plasmids used for lentivirus production.

**Supplementary Figures**

**Supplementary Figure 1:** An example of a CFSE-based proliferation assay.

**Supplementary Figure 2:** TCR clonotype distribution.

**Supplementary Figure 3:** Dose response curve for C-peptide responding cells.

**Supplementary Figure 4:** Validation of the TCR screening assay**.**

**Supplementary Method**

1. **The CFSE-based proliferation assay and 10X analysis**

The CFSE (5,6-carboxylfluorescein diacetate succinimidyl ester) proliferation assays were performed as described previously [6]. Briefly, PBMC from T1D subjects were labelled with 0.1μM CFSE (Life Technologies, Carlsbad, CA) and cultured in presence, or absence, of C-peptide (10 μM) and tetanus toxoid (0.33 LfU/ml). After 7 days of culture labelling buffer (1%BSAin PBS) was used to wash and resuspende~~d~~ the cells in a final volume of 100μl per conditions. 10μl of TruStain FcX (Biolegend) was added to the suspension per each condition then gently mixed and incubated for 10 min at 4°C. Cells were stained with TotalSeq-C anti-human hashtag antibodies (BioLegend #TotalSeq^TM^-C0251, TotalSeq^TM^-C0256) at the final concentration of 1:100 along with anti-human CDA AF647 (clone OKT4). After 30 min of incubation at 4°C cells were washed and kept in Resuspension buffer (0.04% BSA in PBS). CD4^+^ T-cell proliferation was measured by determining the number of CD4^+^~~,~~ CFSE^dim^ cells for every 5,000 CD4^+^ CFSE^bright^ cells. The results are presented as a Cell Division Index (CDI) which is the ratio of the number of CD4^+^ cells that have proliferated in the presence of antigen: without antigen [6]. CD4^+^ CFSE^dim^ cells from both C-peptide and tetanus toxoid treated sample were sorted separately using FACS Aria Fusion (BD). Pre-barcoded sorted cells from both the antigen treatment were mixed at 1:1 ratio and washed before loaded into Chromium Next GEM Chip K as per rest of Chromium Next GEM Single cells 5’v2 Dual index with feature barcode kit according to 10X Genomics protocol.

1. **TCR analysis**

V(D)J and feature barcode libraries were prepared by using the 10X Genomics Chromium Single Cell Immune Profiling Solution Kit (5′ Gene Expression and V(D)J) as per manufacture protocol. The libraries were sequenced following Illumina’s specifications using paired-end sequencing (2 × 150bp) on Illumina NovaSeq system with minimal of ~5,000 reads/cell for V(D)J and feature barcode libraries. The V(D)J reads were aligned to the 10x pre-built reference genome to GRCh38 (GRCh38-2020-A and vdj_GRCh38_alts_ensembl-5.0.0) and quantified using CellRanger multi pipeline (10x Genomics, version 6.0.1). Filtered gene count matrix from CellRanger was analysed by using R (version 4.1.0) and Seurat (version 4.0.5). Cell-specific filtering was performed by retaining cells with RNA features between 200 and 7,500, and less than 5% mitochondrial RNA. Centred log ratio transformation across cells by Seurat’s function NormalizeData was used to normalise raw counts of feature barcode. Expression of hastag antibodies was used to distinguish T cells that had responded to C-peptide or tetanus toxoid, these data were then filtered include only cells that expressed one TRB chains and one or two TRA chains. Clones with two TRA chains will be split as two sister clones harbouring one of the two TRA chain with the shared TRB chain. The TCR sequences of up to the 30 most abundant clonotypes for CD4^+^ T cell population were assembled by using custom R script for DNA synthesis and cloning.

1. **Lentiviral constructs for expressing TCR genes**

The TCR genes expressed by the most abundant TCR clonotypes were cloned into modified versions of pRRLSIN.cPPT.PGK-GFP.WPRE. More specifically, the EGFP gene (from pRRLSIN.cPPT.PGK-GFP.WPRE) was excised by BamHI/SalI digestion and replaced by human TRAC or TRBC2 with either PmeI (TRAC) or SbfI (TRBC2) site at the 5’ end of the TCR constant regions. TCR variable alpha and TCR beta genes were synthesized by IDT as gene blocks and cloned into the modified pRRLSIN vectors by In-fusion cloning (Takara) according to the manufacturer’s protocol. Plasmids were extracted from growing *E. coli* and the inserts were verified by sequencing.

**Supplementary Table 1**: Demographics and HLA of T1D blood donors

| **identifier** | **Sex** | **T1D duration**  **(years)** | **Age** | **HLA Class I alleles** | **HLA Class II alleles** |
| --- | --- | --- | --- | --- | --- |
|  |  |  | **(years)** |  |  |
| 312T4 | F | 14 | 22 | A 11:01, 24:02  B 15:01, 18:01  C 03:03, 05:01 | DRB1*03:01, 04:01  DRB3*02:02 DRB4*01:03  DPB1*02:02, 03:01  DPA1*01:03  DQB1*02:01, 03:02 DQA1*03:01, 05:01 |
| 199T1 | M | 0.12 | 15 | A 11:01  B 35:01  C 04:01 | DRB1*01:01, 15:02  DPB1*04:01, 13:01  DQB1*05:01, 06:01 DQA1*01:01, 01:03 |
| 282T2 | M | 2.10 | 20 | A 01:01, 03:01  B 08:01  C 07:01 | DRB1*03:01  DRB3*01:01, 02:02  DPB1*04:01 DPA1*01:03  DQB1*02:01 DQA1*05:01 |
| 260T1 | M | 0.2 | 14 | A 02:01; 03;01  B 15:01, 38:01  C 03:04, 12:03 | DRB1*03:01, 04:01  DRB3*01:01, DRB4*01:03  DPB1*01:01, *02:01  DPA1*01:03, 02:01  DQB1*02:01, 03:02  DQA1*03:01, 05:01 |
| 590T1 | M | 1.4 | 14 | A 02:01; 03;01  B 08:01, 47:01  C 06:02, 07:01 | DRB1*04:01, 04:05 DRB4*01:03  DPB1*04:01, *17:01  DPA1*01:03, 02:01  DQB1*03:01, 03:02  DQA1*03:03 |

**Supplementary Table 2:** Summary of CFSE assay responses and TCR antigen specificity screening.

*Frozen sample used

C-pept: Full length C-peptide (PI_33-63_)

TT: Tetanus toxoid

| ID | Antigen | CDI | TCR screened | Frequency range | Antigen specific TCR  Hi Low | | % Antigen Specific TCR  (incl Hi and low) |
| --- | --- | --- | --- | --- | --- | --- | --- |
| 312T4 | C-pept | 4.3 | 30 | 126-2 | 1 | 0 | 3.3 |
|  | TT | 398.3 | 15 | 41-13 | 5 | 3 | 53.3 |
|  |  |  |  |  |  |  |  |
| 199T1* | C-pept | 3.1 | 25 | 45-2 | 0 | 0 | 0.0 |
|  | TT | 676.4 | 11 | 27-12 | 8 | 1 | 81.8 |
|  |  |  |  |  |  |  |  |
| 282T2 | C-pept | 6.3 | 25 | 63-3 | 1 | 1 | 8.0 |
|  | TT | 374.5 | 25 | 299-11 | 6 | 6 | 48.0 |
|  |  |  |  |  |  |  |  |
| 260T1 | C-pept | 26.4 | 25 | 15-1 | 1 | 1 | 8.0 |
|  | TT | 211.2 | 25 | 13-2 | 1 | 2 | 12.0 |
|  |  |  |  |  |  |  |  |
| 590T1 | C-pept | 3.5 | 25 | 68-9 | 1 | 0 | 4.0 |
|  | TT | 18.3 | 25 | 329-9 | 5 | 0 | 20.0 |

**Supplementary Table 3:** TCR sequence of C-peptide specific CD4^+^ T-cell clones

| **Donor ID** | **Clonotype**  **ID** | **MSC**  **(μM)** | **EC_50_ (μM)** | **Freq** | **rank** | **tra_cdr3** | **TRAV** | **TRAJ** | **trb_cdr3** | **TRBV** | **TRBD** | **TRBJ** |
| --- | --- | --- | --- | --- | --- | --- | --- | --- | --- | --- | --- | --- |
| 312T1 | 11 | 10 | 34.4 | 7 | 12 | CAVKAHTNAGKSTF | 12-2*01 | 27*01 | CASSWGGKGSGNYGYTF | 5-5*02 | 1*01 | 1-2*01 |
| 282T2 | 3_2 | 10 | 38.5 | 23 | 4 | CAVDITGRGGATNKLIF | 39*01 | 43*01 | CATSRGDPSSSYEQYF | 15*02 | 2*01 | 2-7*01 |
| 282T2 | 9 | 50 | >50 | 15 | 8 | CAVVRNTGKLIF | 8-3*01 | 37*01 | CASSPTTGTGGETQYF | 7-6*01 | 1*01 | 2-5*01 |
| 260T1 | 4 | 2 | 10.75 | 6 | 4 | CAVSVDNAGNMLTF | 8-4*01 | 39*01 | CSAGLAGGGTQYF | 20-1*02 | 2*02 | 2-3*01 |
| 260T1 | 24 | 50 | >50 | 1 | 9 | CAALKGGANNAGKSTF | 13-1*02 | 27*01 | CASSQVGGWTYEQYF | 4-2*01 | 2*01 | 2-7*01 |
| 590T1 | 1 | 0.4 | 2.6 | 64 | 2 | CIVESGANNLFF | 26-1*02 | 36*01 | CASSLGLRLYNEQFF | 7-2*01 | 2*01 | 2-1*01 |

**Supplementary Table 4.** Plasmids used for lentivirus production.

| **Plasmid name** | **Protein encoded** | **Source** |
| --- | --- | --- |
| pRRLSIN.cPPT.PGK-GFP.WPRE | EGFP | A gift from Didier Trono (Addgene plasmid # 12252 ; http://n2t.net/addgene:12252 ; RRID:Addgene_12252). |
| pMDLg/pRRE | HIV Gag, Pol | A gift from Didier Trono (Addgene plasmid # 12251 ; http://n2t.net/addgene:12251 ; RRID:Addgene_12251). |
| pRSV-Rev | Rev | A gift from Didier Trono (Addgene plasmid # 12253 ; http://n2t.net/addgene:12253 ; RRID:Addgene_12253). |
| pMD2.G | VSV-G | A gift from Didier Trono (Addgene plasmid # 12259 ; http://n2t.net/addgene:12259 ; RRID:Addgene_12259). |
| pRRLSIN.cPPT.PGK-TRAC.WPRE | TCR alpha constant region | This paper. |
| pRRLSIN.cPPT.PGK-TRBC.WPRE | TCR beta 2 constant region | This paper. |

**Supplementary Figure 1:** An example of a CFSE-based proliferation assay. Representative FACS plots from a CFSE-based proliferation assay with donor 260T1’s PBMC: no antigen ‘Nil’, tetanus toxoid (TT) and full-length C-peptide (C-pep). Cells within the CD4^+^ CFSE^dim^ gate were sorted and further analysed.

**Supplementary Figure 2** TCR clonotype distribution. The clonotype distribution for the most abundant TCRs tested against each antigen was plotted for all donors. On left side in blue are the Tetanus toxoid responding clones/TCR and right side in red bars are the C-peptide responding clones/TCR. From top to bottom the order (sequence) of the donors is 312T4, 199T1, 282T2, 260T1 and 590T1. TCR Clones were ranked as per abundance, where 1 is more abundant and 25 is least abundant. TCR clone/s with identical beta chain but different alpha chain are denoted as A and B following the clone number.

**Supplementary Figure 3:** C-peptide dose-response curves. The dose-response curves for CD4^+^ T-cell avatar stimulated with C-peptide are shown. A selection of C-peptide specific and non-specific TCRs from each donor were transduced into the Jurka reporter line, JNL. TCR transduced cells were purified to > 90% TCR^+^. These T-cell avatars were tested against titrated concentration of full-length C-peptide (0.016-50mM). the symbols indicate the TCRs tested for each donor. Responses in the absence of any antigen were used to determine the background luciferase signal and calculate the Δluciferase. The dotted line at 2x10^4^ Δluciferase indicates the threshold above which is considered to be a positive response. One representative of at least two experiments is shown.

**Supplementary Figure 4:** Validation of the TCR screening assay**.** Receiver-Operator Characteristic (ROC) curves for antigen-responsive clonotypes were plotted to show the sensitivity and specificity of the screening response. Separate curves were generated for validated responder and non-responder TCR clones for C-peptide (A) and Tetanus toxoid (B). The area under the ROC curve for C-peptide was 1.00, while for Tetanus toxoid it was 0.70
